# Clinical and Radiological Concordance of Bone Tumours Using Histopathology as the Gold Standard: A Pilot Study

**DOI:** 10.7759/cureus.109401

**Published:** 2026-05-21

**Authors:** Varun Vijay, Abhay Vikram Singh, Deepti Mishra, Pradyumn Singh

**Affiliations:** 1 Department of Orthopedic Surgery, Kalyan Singh Superspeciality Cancer Institute, Lucknow, IND; 2 Department of Orthopedic Surgery, Kalyan Singh Superspeciality Cancer Institute,, Lucknow, IND; 3 Department of Pathology and Laboratory Medicine, Kalyan Singh Superspeciality Cancer Institute, Lucknow, IND; 4 Department of Pathology, Dr. Ram Manohar Lohia Institute of Medical Sciences, Lucknow, IND

**Keywords:** bone tumours, clinico-radiological concordance, ewing sarcoma, giant cell tumour, histopathology, osteosarcoma

## Abstract

Background: Bone tumours constitute a heterogeneous group of lesions with overlapping clinical and radiological features, making accurate preoperative diagnosis challenging. Correlating clinical findings and imaging with histopathology may improve diagnostic confidence and guide management. The present study aimed to evaluate the clinical and radiological profile of bone tumours and to determine the degree of clinico-radiological concordance with histopathology, which served as the gold standard.

Materials and methods: This hospital-based pilot observational study included 34 patients with suspected bone tumours who underwent clinico-radiological evaluation followed by histopathological confirmation. Demographic and clinical details were obtained from case records. Radiological assessment was based on plain radiography and MRI, with additional CT and PET-CT as indicated. Lesions were assessed for age predilection, anatomical site, bone segment involved, pattern of destruction, margin, cortical breach, periosteal reaction, matrix mineralisation, and soft-tissue extension. A provisional clinico-radiological diagnosis was assigned in each case. Histopathological diagnosis served as the gold standard. Data were analysed using descriptive statistics and diagnostic performance indices.

Results: The mean age at presentation was 24.14 ± 13.17 years, with the highest incidence in the 10-19-year age group (44.1%). Males accounted for 73.5% of cases. Long bones were affected in 88.2% of cases, the most common being the femur (41.2%). Histopathology revealed malignant tumours in 73.5%, benign tumours in 20.6%, and non-neoplastic or inflammatory lesions in 5.9%. The most frequent final diagnosis was osteosarcoma (47.1%), followed by giant cell tumour (17.6%) and Ewing sarcoma (14.7%). Family-level clinico-radiological concordance was 47.1%, rising to 70.6% when the final diagnosis was included among radiological differentials. The sensitivity, specificity, and overall diagnostic accuracy for detecting malignancy were 84.0%, 77.8%, and 82.4%, respectively.

Conclusion: Clinico-radiological evaluation is valuable for preliminary diagnosis, biological stratification, and narrowing the differential diagnosis of bone tumours; however, histopathology remains indispensable for definitive diagnosis and accurate tumour classification.

## Introduction

Bone tumours constitute a heterogeneous group of lesions, ranging from benign, self-limiting entities to highly aggressive primary malignancies that carry significant morbidity and mortality. Although relatively uncommon compared with other neoplasms, their diagnostic evaluation is often challenging because many lesions share overlapping clinical and radiological features, while their biological behaviour and treatment differ substantially. Recent WHO classifications have further emphasised the need for an integrated diagnosis based on morphology, radiology, and clinicopathological context rather than the isolated interpretation of a single modality [1,2].

In routine practice, the initial assessment of a suspected bone tumour begins with careful clinical evaluation and conventional radiography. Patient age, anatomical site, compartment of origin, pattern of bone destruction, matrix mineralisation, periosteal reaction, cortical breach, and associated soft-tissue component remain key parameters for narrowing the differential diagnosis. Conventional radiography remains the first-line imaging modality for lesion characterisation. At the same time, MRI provides superior delineation of marrow involvement, extra-osseous extension, neurovascular proximity, skip lesions, and internal soft-tissue architecture [3-5]. Thus, clinico-radiological concordance is central to diagnostic stratification and biopsy planning.

Despite advances in imaging, histopathology remains the gold standard for definitive diagnosis of bone tumours. Imaging can strongly suggest the nature and aggressiveness of a lesion. Still, histopathological confirmation is essential in diagnostically equivocal cases, in malignant lesions, and whenever therapeutic decisions depend on precise tumour typing and grading. Prior studies have shown that radiological assessment shows good but imperfect concordance with histopathology, underscoring the value of a multidisciplinary approach that integrates clinical findings, imaging, and tissue diagnosis [6-8]. Institutional studies from South Asia have similarly reported substantial clinico-radiological-pathological agreement, while also highlighting that misclassification may occur when lesions with similar radiographic patterns are encountered [7-11].

Because the spectrum of bone tumours varies across age groups, skeletal sites, and hospital settings, local institutional data remain important for understanding disease patterns and diagnostic performance. A structured analysis comparing clinical presentation and radiological impression with histopathological diagnosis can help identify common tumour types, define frequently involved anatomical sites, and assess the practical reliability of preoperative clinico-radiological diagnosis. In this context, the present pilot study was undertaken to evaluate the clinical and radiological profile of bone tumours and to correlate these findings with histopathology, the gold standard.

## Materials and methods

Study design and setting

This hospital-based observational pilot study was conducted at Kalyan Singh Superspeciality Cancer Institute, a tertiary-level cancer institute in Lucknow, India. The study aimed to evaluate the clinico-radiological profile of suspected bone tumours and correlate these findings with histopathological diagnosis.

Study population

A total of 34 patients with suspected bone tumours were included. All patients underwent detailed clinico-radiological evaluation, followed by histopathological confirmation. Demographic and clinical data were obtained from institutional case records.

Inclusion and exclusion criteria

Only patients with complete clinical, radiological, and histopathological data were included in the study. Patients with incomplete records, previously treated or recurrent lesions, metastatic bone disease, and non-diagnostic histopathology were excluded.

Clinical assessment

Clinical details, including age, sex, presenting symptoms (such as pain, swelling, and functional limitation), and symptom duration, were documented to assess clinicopathological patterns.

Radiological evaluation

All patients underwent conventional radiography and MRI. Lesions were systematically evaluated for age predilection, anatomical site, bone segment involved, pattern of bone destruction, lesion margins, cortical breach, periosteal reaction, matrix mineralisation, and soft-tissue extension. Additional imaging modalities, such as CT and PET-CT, were used in selected cases as indicated.

Clinico-Radiological Diagnosis

Based on clinical and imaging findings, a provisional diagnosis was assigned to each case. Where appropriate, a list of differential diagnoses was also provided. For concordance analysis, the first radiological impression was considered the primary diagnosis.

Histopathological evaluation

Histopathological diagnosis, derived from biopsy or surgical resection specimens, was considered the gold standard. All cases were classified as benign, malignant, or non-neoplastic/inflammatory based on histological findings.

Outcome measures

The primary outcome measure was concordance between the clinico-radiological diagnosis and the final histopathological diagnosis. Concordance was assessed at both the tumour-family and behaviour levels (benign vs. malignant).

Statistical analysis

Data were analysed using descriptive statistics. The diagnostic performance of clinico-radiological assessment was evaluated by calculating sensitivity, specificity, positive predictive value (PPV), negative predictive value (NPV), and overall accuracy. Cohen’s kappa coefficient was used to assess agreement between radiological and histopathological diagnoses.

Ethical considerations

Ethical approval was obtained from the Institutional Ethics Committee of Kalyan Singh Superspeciality Cancer Institute (approval number: 2023-02-IMP-EXP-1). Patient confidentiality was strictly maintained, and all data were anonymised before analysis.

## Results

Patient demographics and anatomical distribution

A total of 34 patients with suspected bone tumours were included in the study. The cohort was predominantly young, with a mean age of 24.14 ± 13.17 years and a median of 19.5 years (range: three to 60 years). The highest case burden was observed in the second decade of life, with 44.1% of patients in the 10-19-year age group, followed by 20.6% in the 20-29-year age group. Together, these two decades accounted for nearly two-thirds of the study population, underscoring the strong predilection of bone tumours for adolescents and young adults. A clear male predominance was evident, with 25 males (73.5%) and nine females (26.5%), corresponding to a male-to-female ratio of 2.8:1. Clinically, pain with or without swelling was the most common presenting complaint, often associated with varying degrees of functional limitation. The anatomical distribution of lesions showed a striking predominance of long-bone involvement, observed in 88.2% of cases. The femur was the most frequently affected bone (41.2%), followed by the tibia (23.5%) and humerus (17.6%), which together accounted for the majority of lesions. Less commonly involved sites included the fibula and pelvis/hip region (5.9% each), while isolated involvement of the ankle/foot and other bones was rare (2.9% each).

Segmental analysis revealed a strong predilection for the metaphyseal regions, reflected in the high proportions of proximal (47.1%) and distal (32.4%) involvement. Diaphyseal lesions constituted a smaller subset (11.8%), while atypical or other locations accounted for 8.8% of cases. This distribution aligns with the known biological behaviour of common primary bone tumours, particularly osteosarcoma and giant cell tumour, which frequently arise in the metaphyseal/epiphyseal regions of long bones around the knee (Table 1).

**Table 1:** Demographic and anatomical profile of the study cohort (n=34).

Variable	Value
Age, mean ± SD (years)	24.14 ± 13.17
Age category	Number of cases (percentage)
<10 years	2 (5.9%)
10-19 years	15 (44.1%)
20-29 years	7 (20.6%)
30-39 years	5 (14.7%)
40-49 years	3 (8.8%)
≥50 years	2 (5.9%)
Gender	Number of cases (percentage)
Male	25 (73.5%)
Female	9 (26.5%)
Bone involved	Number of cases (percentage)
Femur	14 (41.2%)
Tibia	8 (23.5%)
Humerus	6 (17.6%)
Fibula	2 (5.9%)
Pelvis/Hip	2 (5.9%)
Other	1 (2.9%)
Ankle/Foot	1 (2.9%)
Segment of bone involved	Number of cases (percentage)
Proximal	16 (47.1%)
Distal	11 (32.4%)
Diaphyseal	4 (11.8%)
Other	3 (8.8%)

Clinico-radiological assessment and imaging characteristics

**Figure 1:**
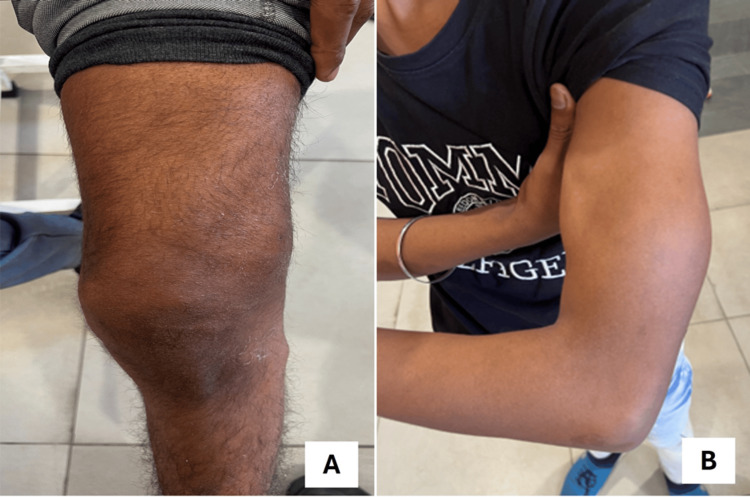
(A) Swelling around the lateral aspect of the lower end of the femur; (B) Swelling around the postero-lateral aspect of left midshaft arm

On initial clinico-radiological evaluation, osteosarcoma was the most common provisional diagnosis (44.1%), reflecting its high prevalence and characteristic imaging features. Indeterminate malignant or expansile lesions accounted for 17.6% of cases, indicating diagnostic uncertainty among patients with overlapping imaging patterns. Ewing sarcoma was suggested in 11.8% of cases, while chondrosarcoma and giant cell tumour were each proposed in 8.8% of cases (Figure 1). Isolated cases were categorised as aneurysmal bone cysts, benign non-specific lesions, and synovial/fibrosarcomas.

A notable finding was the frequent use of multiple differential diagnoses, documented in 64.7% of cases, with a mean of 2.14 ± 1.11 differentials per case. This reflects the inherent complexity of bone tumour imaging, where overlapping radiographic features often necessitate a probabilistic diagnostic approach rather than a single definitive label.

**Figure 2:**
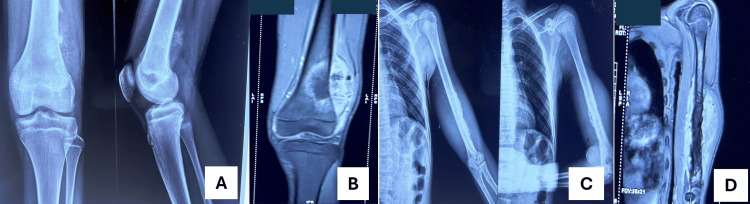
(A) X-ray showing cortical destruction of the metaphyseal region of the lower end of the femur with a sunburst appearance; (B) MRI of the lower end of the femur showing a permeative lesion with mixed lysis and sclerosis and periosteal reaction; (C) X-ray showing cortical destruction involving the diaphysis of the left humerus. (D) MRI of the left humerus showing a destructive intramedullary bone lesion with a large adjacent soft tissue mass with a cortical breach

X-ray and MRI were the cornerstones of imaging evaluation and were performed in all cases, enabling detailed assessment of marrow involvement, cortical integrity, soft-tissue extension, and neurovascular relationships (Figure 2).

Additional imaging modalities were used selectively, with CT performed in 29.4% of cases and PET-CT in 23.5% of cases, primarily for further characterisation or staging. The most common primary clinico-radiological diagnosis was osteosarcoma in 44.1% cases (n=15), an indeterminate malignant/expansile lesion in 17.6% cases (n=6) and Ewing's sarcoma in 11.8% cases (n=4) (Table 2).

**Table 2:** Primary clinico-radiological diagnosis

Primary clinico-radiological diagnosis	n (%)
Osteosarcoma	15 (44.1%)
Indeterminate malignant/expansile lesion	6 (17.6%)
Ewing sarcoma	4 (11.8%)
Chondrosarcoma	3 (8.8%)
Giant cell tumor	3 (8.8%)
Aneurysmal bone cyst	1 (2.9%)
Benign non-specific	1 (2.9%)
Synovial/fibrosarcoma	1 (2.9%)
Cases with >1 radiological differential diagnosis	22 (64.7%)
Mean number of radiological differentials/case	2.14 ± 1.11

Histopathological spectrum and tumour behaviour

**Figure 3:**
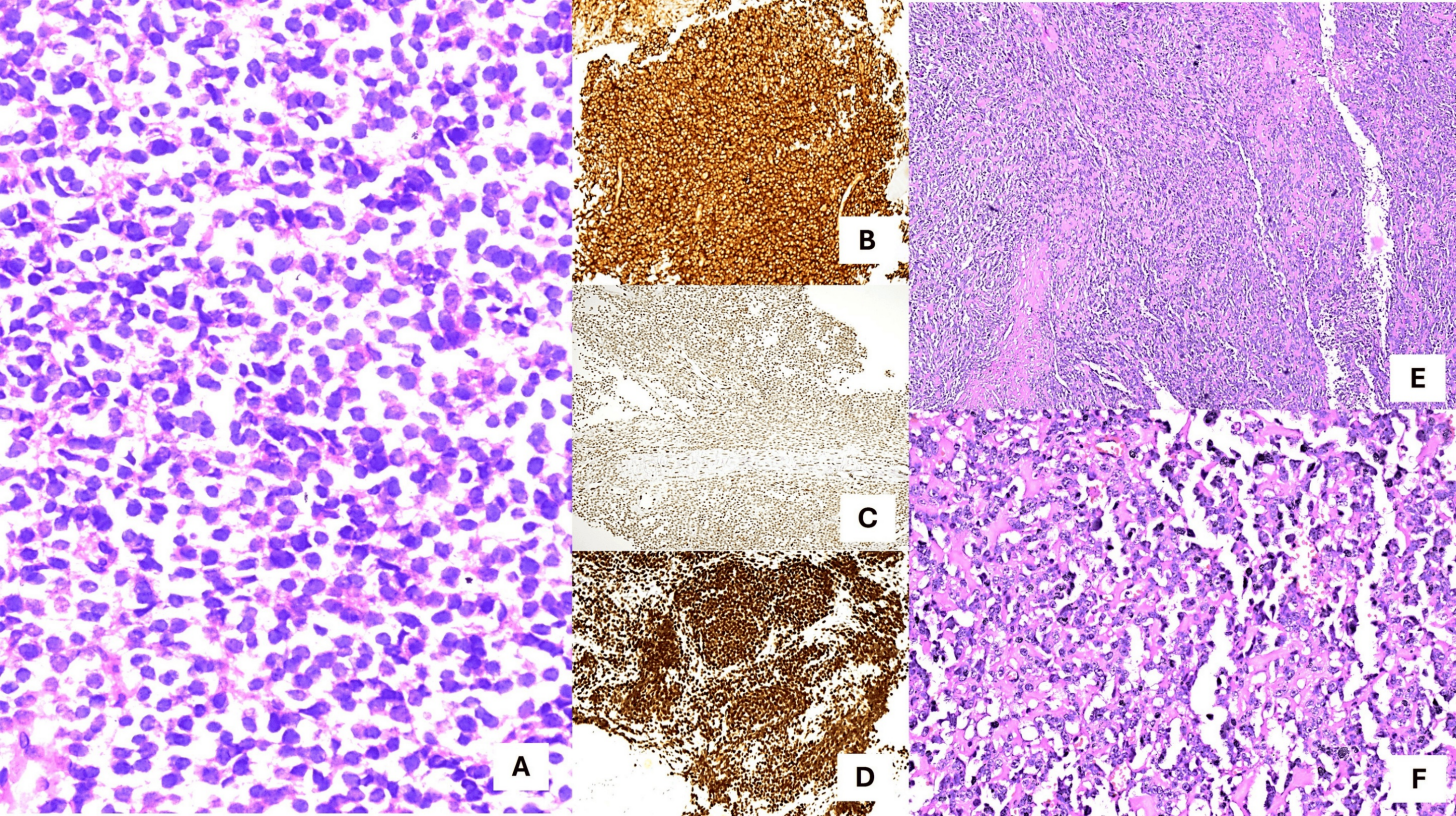
(A-D) Histopathological photomicrograph of a malignant small round cell tumour composed of monotonous cells with scant cytoplasm, round nuclei, and stippled chromatin. The tumour displays diffuse positivity for CD99 (B) nuclear expression for FLI-1 (C) and strong nuclear positivity for NKX2.2 (D), reported as Ewing’s sarcoma (A: haematoxylin and eosin x100, B-D=DAB x100). (E-F): Histopathological photomicrographs from femur growth with a tumour composed of pleomorphic cells having hyperchromatic nuclei with intervening lacy osteoid are reported as osteosarcoma (E=haematoxylin and eosin x100, F=haematoxylin and eosin x200).

Histopathological examination confirmed that malignant tumours constituted the majority of cases (73.5%), with benign tumours accounting for 20.6% and non-neoplastic/inflammatory lesions for 5.9%. Osteosarcoma was the dominant tumour type, representing nearly half of all cases (47.1%). Giant cell tumour was the second most common lesion (17.6%), followed by Ewing sarcoma (14.7%). Chondrosarcoma accounted for 8.8% of cases, while aneurysmal bone cyst and synovial sarcoma were rare, with one case each (2.9% of cases) (Figure 3).

This distribution highlights the predominance of high-grade malignant lesions in the study cohort, consistent with the tertiary referral setting. The relative frequency of osteosarcoma and Ewing sarcoma further explains the younger age profile observed in the study population (Table 3).

**Table 3:** Histopathological diagnosis and tumour behaviour.

Primary clinico-radiological diagnosis	n (%)
Osteosarcoma	15 (44.1%)
Indeterminate malignant/expansile lesion	6 (17.6%)
Ewing sarcoma	4 (11.8%)
Chondrosarcoma	3 (8.8%)
Giant cell tumor	3 (8.8%)
Aneurysmal bone cyst	1 (2.9%)
Benign non-specific	1 (2.9%)
Synovial/fibrosarcoma	1 (2.9%)
Cases with >1 radiological differential diagnosis	22 (64.7%)
Mean number of radiological differentials/case	2.14 ± 1.11

Clinico-radiological and histopathological concordance

Correlation between the primary clinico-radiological impression and the final histopathological diagnosis showed exact concordance in 47.1% of cases. However, when concordance was broadened to include cases in which the final diagnosis was within the radiological differential list, agreement increased substantially to 70.6%. This marked improvement underscores the practical value of radiological differentials in capturing diagnostic possibilities, even when the primary impression is not definitive.

At the tumour-behaviour level, concordance in classifying tumours as malignant versus benign/non-neoplastic was observed in 73.5% of cases. Cohen’s kappa analysis showed fair agreement for tumour-type classification (κ = 0.295) and moderate agreement for behaviour-level classification (κ = 0.440).

Tumour-specific analysis revealed variable concordance across diagnostic categories. Osteosarcoma showed relatively high exact concordance (62.5%), reflecting its more characteristic imaging profile. Chondrosarcoma showed similar concordance (66.7%), though based on fewer cases. In contrast, Ewing sarcoma exhibited low exact concordance (20.0%), highlighting the diagnostic challenges associated with small round-cell tumours, which often share imaging features with other aggressive lesions. No exact concordance was observed for aneurysmal bone cyst, synovial sarcoma, or non-neoplastic/inflammatory lesions, although some of these were included in broader differential diagnoses (Table 4).

**Table 4:** Concordance between clinico-radiological impression and final histopathology

Final histopathological diagnosis	Exact concordance (between clinico-radiological and histopathological diagnosis; Number of cases/Total cases (percentage))	Differential concordance (between clinico-radiological and histopathological diagnosis; Number of cases/Total cases (percentage))
Aneurysmal bone cyst	0/1 (0.0%)	1/1 (100.0%)
Chondrosarcoma	2/3 (66.7%)	3/3 (100.0%)
Ewing sarcoma	1/5 (20.0%)	4/5 (80.0%)
Giant cell tumor	3/6 (50.0%)	3/6 (50.0%)
Non-neoplastic/inflammatory	0/2 (0.0%)	1/2 (50.0%)
Osteosarcoma	10/16 (62.5%)	12/16 (75.0%)
Synovial sarcoma	0/1 (0.0%)	0/1 (0.0%)
Exact family-level concordance	16/34 (47.1%)	—
Differential includes the final diagnosis	24/34 (70.6%)	—
Behaviour-level concordance	25/34 (73.5%)	—
Cohen κ (family-level)	0.295	—
Cohen κ (behaviour-level)	0.44	—

Diagnostic performance for the identification of malignancy

When evaluated for its ability to distinguish malignant from non-malignant lesions, the clinico-radiological assessment demonstrated strong diagnostic performance. Sensitivity was 84.0%, indicating that the majority of malignant tumours were correctly identified on initial evaluation. Specificity was 77.8%, reflecting a moderate ability to correctly classify non-malignant lesions.

The PPV was high (91.3%), indicating that lesions identified as malignant on imaging were highly likely to be confirmed as malignant on histopathology. In contrast, the NPV was lower (63.6%), indicating that a subset of lesions initially considered non-malignant were subsequently found to be malignant. The overall diagnostic accuracy was 82.4%, supporting the clinical utility of combined clinico-radiological assessment for initial tumour stratification.

Of the 25 histopathologically confirmed malignant cases, 21 (84.0%) were correctly identified, while four cases were misclassified. Among the nine non-malignant cases, seven were correctly categorised, and two were incorrectly labelled as malignant (Table 5).

**Table 5:** Diagnostic performance of the primary clinico-radiological impression for malignancy. *The non-malignant category includes benign tumours and non-neoplastic/inflammatory lesions.

Primary clinico-radiological impression	Histopathology malignant	Histopathology non-malignant
Radiology malignant	21	2
Radiology non-malignant^*^/ indeterminate	4	7
Sensitivity	84.00%	—
Specificity	77.80%	—
Positive predictive value	91.30%	—
Negative predictive value	63.60%	—
Overall accuracy	82.40%	—

Pattern and analysis of discordant cases

A total of 18 cases (52.9%) showed discordance between the primary clinico-radiological impression and the final histopathological diagnosis at the tumour-family level. Analysis of these discordant cases revealed specific patterns of misclassification.

The most frequent discrepancy was radiological osteosarcoma subsequently diagnosed as Ewing sarcoma, observed in 16.7% of discordant cases. Another common pattern was indeterminate malignant or expansile lesions ultimately confirmed as osteosarcoma (16.7%), reflecting diagnostic uncertainty in lesions with aggressive but non-specific imaging features.

Additional discordances included Ewing sarcoma misclassified as osteosarcoma (11.1%) and indeterminate lesions later identified as giant cell tumour (11.1%). Other less frequent patterns involved transitions among benign, malignant, and non-neoplastic categories, including cases initially labelled benign or indeterminate that were later diagnosed as malignant on histopathology.

These findings highlight the considerable overlap in imaging characteristics among bone tumours, particularly between osteosarcoma, Ewing sarcoma, and other aggressive or expansile lesions. They also underscore the limitations of imaging for definitive tumour classification and the critical role of histopathological confirmation (Table 6).

**Table 6:** Summary of discordant diagnostic transitions (n=18 discordant cases).

Primary clinico-radiological diagnosis	Final histopathological diagnosis	n (%) among discordant
Osteosarcoma	Ewing sarcoma	3 (16.7%)
Indeterminate malignant/expansile lesion	Osteosarcoma	3 (16.7%)
Ewing sarcoma	Osteosarcoma	2 (11.1%)
Indeterminate malignant/expansile lesion	Giant cell tumor	2 (11.1%)
Ewing sarcoma	Non-neoplastic/inflammatory	1 (5.6%)
Chondrosarcoma	Osteosarcoma	1 (5.6%)
Benign non-specific	Aneurysmal bone cyst	1 (5.6%)
Aneurysmal bone cyst	Ewing sarcoma	1 (5.6%)
Indeterminate malignant/expansile lesion	Non-neoplastic/inflammatory	1 (5.6%)
Osteosarcoma	Chondrosarcoma	1 (5.6%)
Osteosarcoma	Giant cell tumor	1 (5.6%)
Fibro sarcoma	Synovial sarcoma	1 (5.6%)

## Discussion

The present pilot study demonstrated that bone tumours in our cohort were predominantly seen in younger patients, with a mean age of 24.14 years and a peak in the second decade of life, along with a clear male predominance. This pattern is broadly consistent with prior institutional and epidemiological studies. Azad et al. (2022) [7] reported a mean age of 30.5 years in patients evaluated with X-ray and MRI, while Mallikarjunaiah et al. (2025) [10] observed that the most common age group was 11-20 years, with male predominance. Similarly, Piparsania et al. (2025) [9] found a mean age of 29.4 years and a male preponderance, and Jain et al. (2011) [12] also reported that primary bone tumours were most frequent in the 11-20-year age group. The younger age profile in the present series is likely related to the relatively high proportion of osteosarcoma and Ewing sarcoma, both of which are well known to cluster in childhood, adolescence, and early adult life. Ottaviani and Jaffe (2009) [13] and Misaghi et al. (2018) [14] likewise described the classic adolescent peak of osteosarcoma. 

Anatomically, long bones accounted for the vast majority of lesions in our series, with the femur the most common site, followed by the tibia and humerus. This distribution closely parallels previous reports. Mallikarjunaiah et al. (2025) [10] found the femur and tibia to be the two most frequently involved bones, and Piparsania et al. (2025) [9] likewise identified the femur as the leading site. In the Tanzanian study by Said et al. (2024) [8], the femur was also the most commonly affected long bone, while Jain et al. (2011) [12] reported the distal femur as the most common location, followed by the proximal tibia/fibula. The site pattern in the present study also aligns with established tumour biology, as osteosarcoma typically arises in the metaphyseal regions of long bones, especially around the knee, and giant cell tumour characteristically involves the epiphyseal-metaphyseal ends of long bones in young adults. Misaghi et al. (2018) [14] emphasised osteosarcoma's predilection for the distal femur and proximal tibia, whereas Brito et al. (2021) [15] highlighted the around-the-knee predominance of giant cell tumour. 

Regarding the histopathological spectrum, osteosarcoma was the most common final diagnosis in the present study, followed by giant cell tumour and Ewing sarcoma. This aligns with several comparative studies. Azad et al. (2022) [7] concluded that the combination of X-ray and MRI achieved high diagnostic accuracy in a cohort in which lytic and sclerotic matrix patterns were systematically assessed against histopathology. Mallikarjunaiah et al. (2025) [10] reported osteosarcoma as the leading malignant lesion and giant cell tumour as the most frequent benign lesion, findings that are very similar to the present series. Said et al. (2024) [8] also found osteosarcoma to be the most common tumour type on both radiological and histological assessments. By contrast, Piparsania et al. (2025) [9] observed that benign tumours were more common overall, with osteochondroma as the dominant benign lesion, although osteosarcoma remained the leading malignant tumour. This difference from our study likely reflects variations in case mix, referral pathways, and inclusion criteria; our cohort appears enriched for biopsy-proven aggressive lesions that were referred for advanced imaging and specialist evaluation. Jain et al. (2011) [12] similarly reported that benign tumours were more frequent overall in their larger hospital-based review, again underscoring that the tumour spectrum in a pilot, tertiary referral cohort may differ from that of broader pathology registries. 

The imaging-pathology correlation in our study warrants particular emphasis. Exact family-level concordance between the primary clinico-radiological impression and histopathology was modest. Still, concordance improved substantially when the final histological diagnosis was accepted as present anywhere within the radiological differential list. In practical terms, this indicates that imaging was often directionally correct in identifying lesion aggressiveness and narrowing the diagnostic field, even when it did not provide a single exact label. This pattern is clinically plausible and supported by the literature. Miller (2008) [3] stressed that radiographic diagnosis of bone tumours depends on structured assessment of age, location, margins, periosteal reaction, matrix mineralisation, and soft-tissue component rather than on any single sign. Goyal et al. (2019) [4] and Alyas et al. (2007) [5] similarly emphasised that MRI adds value primarily through assessment of marrow extent, cortical breach, extra-osseous extension, and relationship to adjacent structures, thereby refining, but not replacing, histological diagnosis. 

Compared with published concordance studies, the exact agreement in our cohort was lower than that reported in larger series, yet diagnostic performance for identifying malignancy remained clinically meaningful. Piparsania et al. (2025) [9] found radiological and histopathological concordance in 86.7% of cases, with a kappa of 0.78, while Mallikarjunaiah et al. (2025) [10] reported 85% correlation. Patil et al. (2020) [11] similarly demonstrated substantial agreement, with a kappa of 0.749 between clinico-radiological and histopathological diagnoses. Said et al. (2024) [8] reported a sensitivity of 92.1%, a specificity of 73.3%, and an accuracy of 82% for plain radiography in long-bone tumours, and Lange et al. (2016) [6] also showed that imaging methods can achieve meaningful diagnostic accuracy when judged against pathology-proven skeletal lesions. In the present study, sensitivity for malignancy was high, and overall accuracy was good, suggesting that the main limitation was not a failure to recognise aggressive disease but rather overlap among specific tumour families, such as osteosarcoma, Ewing sarcoma, and giant cell-rich or expansile lesions. 

The most common discordant patterns in our data were radiological osteosarcoma that was later proved to be Ewing sarcoma (Figure 3) and indeterminate malignant/expansile lesions that were subsequently diagnosed as osteosarcoma (Figure 4). These discrepancies are understandable because osteosarcoma and Ewing sarcoma may both present in adolescents with pain, aggressive bone destruction, periosteal reaction, cortical interruption, and a soft-tissue mass. Hesla et al. (2021) [16] noted that Ewing sarcoma is a highly aggressive tumour of children and adolescents, commonly involving the femur, tibia, and humerus, sites that overlap with osteosarcoma. Likewise, Misaghi et al. (2018) [14] described osteosarcoma as a rapidly growing tumour of the long bones around the knee in adolescent patients. Such overlap underscores the importance of correlating imaging morphology with age, exact skeletal segment, and histopathology, especially in small round-cell and osteoid-producing lesions. 

Another notable observation in the present cohort was the occurrence of giant cell tumours in young adults, usually around the knee, and partial radiology-pathology mismatch in some cases. This mirrors known diagnostic challenges. Brito et al. (2021) [15] and Jha et al. (2023) [17] described giant cell tumour as a locally aggressive tumour that most often affects the epiphyseal-metaphyseal region of long bones around the knee in patients in the third to fourth decades, but they also emphasised overlap with aneurysmal bone cystic change and other expansile osteolytic lesions. In our series, some lesions initially interpreted as giant cell tumours or non-specific expansile lesions were ultimately classified differently on histopathology, underscoring why tissue diagnosis remains indispensable even when imaging strongly suggests a benign-aggressive lesion. 

Taken together, the present study supports the view that clinico-radiological assessment is highly valuable for lesion localisation, biological stratification, and biopsy planning, but that it is best understood as a probabilistic rather than an absolute diagnostic tool. The study’s lower exact tumour-family concordance compared with larger published series likely reflects its small sample size, pilot design, inclusion of diagnostically complex referral cases, and the fact that multiple differentials were documented in nearly two-thirds of patients. Nevertheless, the high yield in identifying malignant behaviour and the substantial rise in concordance when radiological differentials were considered collectively are clinically important findings. This interpretation aligns with the conclusions of Azad et al. (2022) [7], Said et al. (2024) [8], Patil et al. (2020) [11], Piparsania et al. (2025) [9], and Mallikarjunaiah et al. (2025) [10], all of whom emphasised that radiology and histopathology are complementary and that histopathological confirmation remains essential before definitive treatment. 

Limitations

The small sample size (n=34) limits statistical power and the generalisability of the findings. As a single-centre study conducted at a tertiary care cancer institute, it is likely to suffer from referral bias, with over-representation of complex and malignant cases that may not reflect the broader population. The limited number of certain tumour types restricted detailed subgroup analysis. Concordance assessment based solely on the primary radiological impression may underestimate diagnostic accuracy when multiple differentials were considered. Variability in the use of additional imaging modalities and the lack of assessment of interobserver variability may also influence results.

## Conclusions

In this pilot study, clinicoradiological evaluation showed good overall agreement with histopathology and was particularly useful for narrowing the differential diagnosis based on age, site, pattern of bone destruction, matrix mineralisation, cortical breach, and soft-tissue extension; however, histopathology remained the definitive standard for final classification and treatment planning. The study also reaffirmed the typical profile described in published series, with a predominance of lesions in younger patients, frequent involvement of the long bones around the knee, and pain, with or without swelling, as the commonest presentation. Taken together, these findings support a multidisciplinary diagnostic approach in which clinical assessment, plain radiography, and MRI are interpreted in concert, with all potentially significant or aggressive lesions undergoing tissue confirmation before definitive management. Despite the small sample size, the present data highlight the practical value of structured clinicoradiological-pathological concordance in improving diagnostic confidence, reducing misclassification, and strengthening decision-making in bone tumour care.
